# Successful Endovascular Repair of a Ruptured Popliteal Artery Aneurysm: A Case Report and Literature Review

**DOI:** 10.1155/2020/8745780

**Published:** 2020-03-16

**Authors:** Duncan Muir, Sachin R. Kulkarni

**Affiliations:** ^1^University of Bristol Medical School, UK; ^2^Gloucestershire Hospitals NHS Foundation Trust, Cheltenham, UK

## Abstract

*Introduction*. It is rare for a popliteal artery aneurysm (PAA) to present with rupture. This case reports a longer-term success in the management of a large ruptured popliteal artery aneurysm with an endovascular approach, with a literature review of management of such cases. *Case Report*. An 80-year-old man presented to the accident and emergency department with pain and swelling behind the left knee and at the back of the thigh. An ultrasound scan and subsequent CT angiogram revealed a large 9.4 cm ruptured PAA. The patient had significant comorbidities deeming him unfit for a major surgical intervention of drainage of haematoma and exclusion bypass. Therefore, he underwent urgent endovascular treatment of the ruptured PAA with a covered stent graft. A follow-up duplex scan at 1 year showed a patent stent with no evidence of endoleak, and the patient remained asymptomatic. A clinical follow-up at 18- and 24-month postprocedure showed a patent stent graft and complete resolution of haematoma. *Conclusion*. Whilst open repair with exclusion bypass may still be a treatment of choice, an endovascular approach is both safe and effective in the management of a ruptured PAA in an unfit patient with an acceptable longer-term outcome.

## 1. Introduction

Popliteal artery aneurysms constitute the most prevalent form of peripheral arterial aneurysms, accounting for approximately 85% of all peripheral aneurysms [1]. PAAs carry a high risk of intravascular thrombosis as well as distal embolization causing critical limb ischaemia [1]. They are often asymptomatic; however, they may present with foot ischaemia, leg oedema, and pain behind the knee due to their proximity to nerves. PAA rupture is a rare but recognized presentation that requires a rapid diagnosis and treatment for limb salvage [1]. The standard treatment for such ruptures is a surgical exclusion bypass; however, a number of other management options are described in the literature [2–13]. Such options include endovascular treatment with coil embolization [10] and/or stent graft. Surgical treatment may have an additional benefit of drainage of haematoma preventing its complications as well as restoring the blood supply to the foot. However, in unfit patients, this may not be a viable option. This case report describes a successful management of ruptured PAA by an endovascular technique with a patent stent graft after 24 months in a patient unfit for major surgical intervention.

## 2. Case Presentation

An 80-year-old man presented with a 4-day history of swelling and pain behind the left knee with reduced mobility with very short distance intermittent claudication affecting the left calf and reduced range of knee movement. This patient in the preceding week was admitted under the care of medical specialty where a diagnosis of a spontaneous haematoma behind the thigh was made. Haematoma was thought to be a result of the patient being on oral anticoagulation (warfarin) for atrial fibrillation. The patient did not report history of trauma. He looked frail on the current admission on the vascular ward requiring support for some activities at home and had been on treatment for prostate cancer. He had previously also had coccygectomy for metastasis as well as a recent history of permanent pacemaker insertion for syncopal episodes and bradycardia. He also suffered from hypertension and was on a number of medications including warfarin. He was a lifelong nonsmoker and lived in a retirement home. There was no family history of aneurysms.

Examination revealed extensive bruising at the back of the left thigh and knee with palpable femoral and pedal pulses. The popliteal pulse was difficult to feel due to a large haematoma. The left foot was viable with fully intact sensory and motor function. There was no clinical evidence of calf compartment syndrome on the left leg. The right foot was warm with all palpable pulses in the leg.

**Figure 1:**
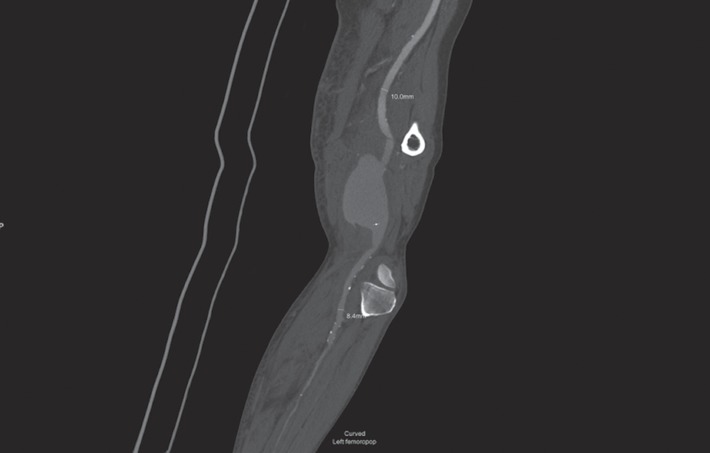
Initial CT angiogram.

An initial ultrasound scan revealed PAA with no evidence of popliteal or femoral vein DVT. A CT angiogram performed on the same day showed generalized arteriomegaly and the ruptured 9.4 cm PAA involving only the above-knee popliteal artery (Figure 1) and relatively disease-free 3-vessel runoff to the ankle and foot. Options to treat this patient were either a surgical exclusion bypass with evacuation of haematoma or an endovascular treatment. For a number of reasons including patient frailty, associated comorbidities, and potential feasibility of successfully treating the PAA with a stent graft due to intact 3-vessel crural runoff, decision was made to treat the aneurysm by an endovascular approach.

**Figure 2:**
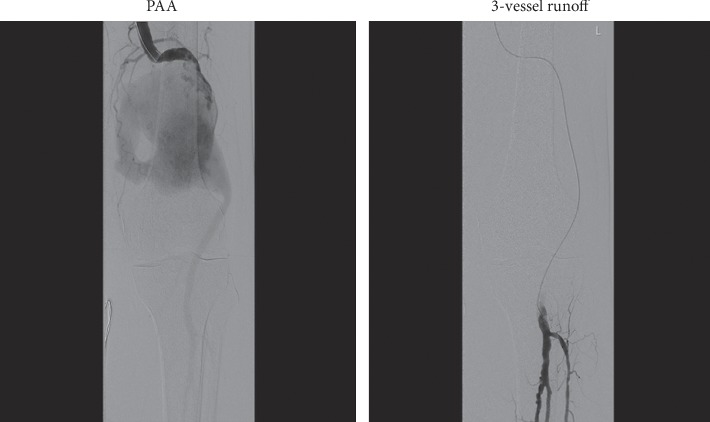
Intraoperative arteriogram.

**Figure 3:**
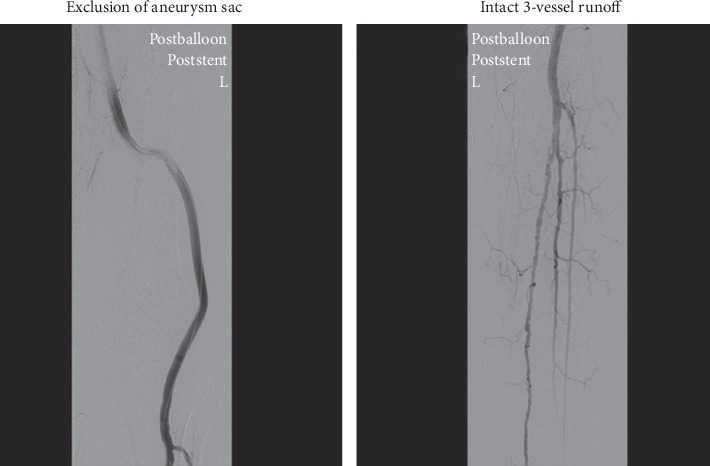
Post-stent-graft insertion.

Under general anaesthesia, the left common femoral artery was explored with a small open incision. An initial angiogram through a 5F sheath confirmed the CT findings of ruptured PAA with 3-vessel runoff (Figure 2). The aneurysm sac was traversed using a curved catheter and a soft hydrophilic wire which was then exchanged to a stiff wire. A 5F sheath was exchanged to an 11F sheath to allow for insertion of 2 Viabahn (Gore®) covered stent grafts of 8-250 mm and 10-100 mm dimensions with a 4 cm overlap which were postdilated with 8 and 10 mm balloons. Postcompletion angiography revealed complete exclusion of the aneurysm sac with improved filling of the runoff vessels (Figure 3). The procedure lasted for approximately 55 minutes.

**Figure 4:**
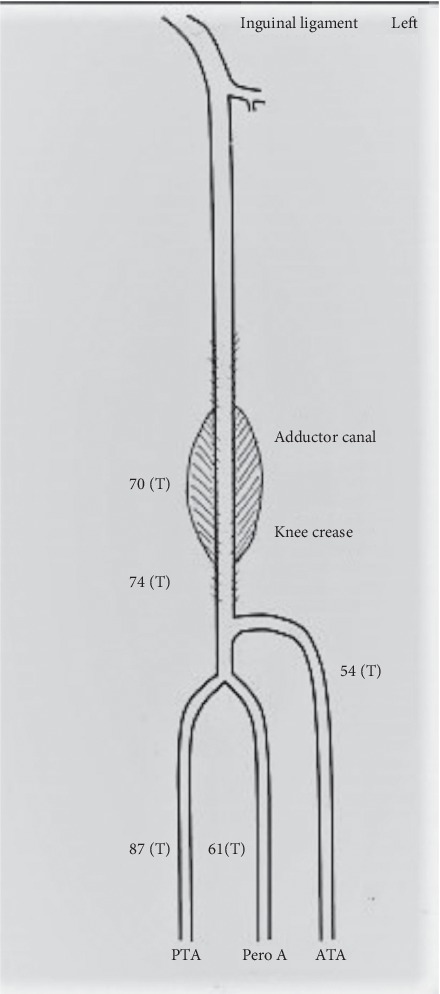
1-year follow-up duplex scan showing the patient's stent. 3-vessel runoff with triphasic flow.

The patient made a good recovery following the repair with a predischarge duplex scan demonstrating a successful exclusion of the aneurysm with good 3-vessel runoff. The patient was discharged on dual antiplatelet therapy which was continued for 6 weeks after which the patient continued on one antiplatelet and an anticoagulant treatment for AF. Subsequent 3-month surveillance duplex scans for up to a year showed a patent stent with intact distal circulation (Figure 4). Duplex surveillance was discontinued after 1 year as per local protocol; a clinical follow-up after 18 and 24 months confirmed a patent stent graft with palpable pedal pulses.

## 3. Discussion

This case reports successful endovascular stent graft repair for exclusion of a ruptured PAA with a patent stent graft after 24 months. It has one of the longest follow-ups with a successful outcome reported in the literature for the treatment of ruptured PAA. Rupture of a PAA is a rare clinical presentation, and literature regarding optimum management is limited, being mostly documented in the form of case reports. The patient in this case report had significant comorbidities and was frail for a surgical repair with exclusion bypass and therefore had an endovascular repair. Open cutdown of the groin rather than a percutaneous approach was performed due to nonavailability of an appropriate closure device at the time of the procedure to close the arterial puncture made for the insertion of the 11F sheath. However, in an ideal situation, the procedure would have been performed percutaneously under local anaesthesia. The patient made an uneventful recovery, and the stent graft was patent after a 24-month follow-up.

A literature review conducted at the time of submission of this case report showed 62 [2–13] reported cases of ruptured PAA. Most cases reported were males, with an age range of 55-96 and a mean age of 81. Different treatment modalities were used to treat such cases; however, the majority of the patients were treated with a standard open repair with exclusion of the aneurysm and bypass surgery. Only one case was treated with coil embolization [10], and twelve were treated by endovascular intervention [2–5, 7, 11–13]. Until recently, open surgical repair has been the approach of choice; however, endovascular repair has become increasingly popular due mostly to availability of better stent grafts with a potentially better longer-term outcome. In a similar case report, the benefit of endovascular repair in a mycotic PAA was described [3]. Extensive comorbidity and sepsis resulted in the patient being considered unfit for open repair, and therefore, an endovascular stent graft was inserted via the ipsilateral femoral artery. One week later, the patient developed an infected haematoma; however, this resolved, and the patient was eventually discharged home. Follow-up CT scans demonstrated no evidence of recurrent infection and satisfactory stent graft function. This case highlights the benefit of endovascular repair in elderly, critically ill, or septic patients.

Endovascular repair is also advocated in another case study in a patient unfit for major surgical repair [4]. In this case report, endovascular stenting was performed in a 96-year-old gentleman with a ruptured PAA. A follow-up after fifteen weeks demonstrated a patent stent graft on Doppler ultrasound and palpable pedal pulses. Literature review at the time of publication of this study concludes that whilst endovascular repair is both safe and effective, open surgical repair still remains a first-choice treatment for PAAs. Therefore, although some of the published data favor endovascular treatment, an open surgical approach is still advocated for an improved longer-term outcome for PAAs. Trinidad-Hernandez et al. [13] reviewed 25 patients (31 limbs) treated with endovascular PAA repair of which 12 limbs were treated with emergency repair (1 ruptured and 11 thrombosed) and the rest with elective repair. Whilst endovascular repair was reportedly better for elective PAAs, major adverse events were higher in limbs that had endovascular repair as an emergency. An observational study by Coskun et al. [7] on 7 patients with ruptured PAAs supports open surgical repair to be the treatment of choice. However, these studies were not comparative studies, and therefore, a prospective randomized study for emergency presentation of PAAs may be required to better understand the treatment options.

## 4. Conclusion

PAA rupture is a rare, potentially limb- and life-threatening condition. On account of the small number of reported cases with relatively short-term outcome data, there is little substantiated evidence to prove which technique of repair has the best outcome. However, there is some evidence suggesting that in unfit elderly patients, an endovascular approach is both safe and effective in the treatment of ruptured PAAs.
